# Determination of Maximum Oil Yield, Quality Indicators and Absorbance Spectra of Hulled Sunflower Seeds Oil Extraction under Axial Loading

**DOI:** 10.3390/foods11182866

**Published:** 2022-09-16

**Authors:** Abraham Kabutey, David Herák, Čestmír Mizera

**Affiliations:** Department of Mechanical Engineering, Faculty of Engineering, Czech University of Life Sciences Prague, 165 20 Prague, Czech Republic

**Keywords:** edible oilseeds, oil recovery, heating temperatures, laboratory production, quality usage

## Abstract

The present study aims to estimate the maximum oil yield of hulled sunflower seed samples in a uniaxial process under a load of 40 kN and speed of 4 mm/min. The oil samples were assessed for their quality parameters and spectra curves within the wavelength range of 325–600 nm. The results show that heating temperatures in the range of 40 °C to 80 °C increased the oil output; however, a maximum oil yield of 48.869 ± 6.023% with a minimum energy of 533.709 ± 65.644 J at the fifth repeated pressing was obtained from the unheated sample compared to the heated samples. The peroxide values ranged from 6.898 ± 0.144 to 7.290 ± 0.507 meq O_2_/kg, acid values from 1.043 ± 0.166 to 1.998 ± 0.276 mg KOH/g oil and free fatty acid values from 0.521 ± 0.083 to 0.999 ± 0.138 mg KOH/g oil, which were within the recommended quality threshold. There were significant spectral differences among the oil samples. A single absorbance peak was observed at 350 nm for all oil samples, indicating low levels of pigment molecules in the oil. The study revealed the need for repeated pressings to recover the considerable residual oil remaining in the seedcake after the first pressing.

## 1. Introduction

Edible/vegetable oil consumption is mainly based on palm, soybean, rapeseed and sunflower oil [1,2]. In 2020/2021, palm and soybean were the most utilized vegetable fats worldwide with 38.4% and 34.6% of the vegetable oil market with a consumption rate of 75.45 and 59.48 million metric tons, respectively. In addition, sunflower and rape oils were exploited at 9.7% and 6.3% with a consumption rate of 19.02 and 27.64 million metric tons, respectively [3,4,5]. According to Ng et al. [6] and Rifna et al. [7], worldwide vegetable oil consumption is projected to be above 170 million tons annually. Globally, sunflower is the third most important oilseed crop after soybean and rapeseed. The crop produces oil for food for human consumption and meal for animal feed as well as for industrial applications such as biodiesel and lubricants [5,8,9]. Vegetable oil can be used as a therapeutic for Alzheimer’s disease [10,11] and prevention of ultraviolet radiation on the human skin [12].

Oilseed extraction techniques affect oil yield and quality [13,14]. Large-scale oil production from oil-bearing crops such as sunflower usually integrates both mechanical pressing and solvent extraction operations [15]. Mechanical screw pressing is the most used for oilseed pressing; however, its efficiency is low in terms of oil output, leaving a higher amount of the residual oil in the seed/press cake [16,17,18]. Several factors affect the mechanical screw press, including press geometry (screw and barrel), operating conditions (such as pressure) and seed pretreatments (such as temperature at the screw inlet, press cylinder and press head) [15,19,20,21].

The pressure developed in a screw press is rather difficult to control and predict [18]. In a uniaxial test by using a piston and a compression machine at a given pressure and speed, the oil-bearing material is put into a pressing vessel with holes at the bottom that allow the oil to escape while the press/seedcake is contained [22,23,24]. This process can be used to determine the required pressure/energy for obtaining the maximum oil yield from the bulk oil-bearing material and the residual oil in the press cake. The maximum oil yield is achieved at the first pressing, while the residual oil is gained through repetitive loading until no further pressing of the material is required (plastic deformation). The deformation energy is calculated from the area under the force–deformation curve according to the trapezoidal rule [22,25]. Compression factors (speed, force, vessel diameter and pressing height) and material pretreatments (heating temperature and heating time) thus influence oil recovery efficiency, residual oil in the press cake and the energy requirement [26,27].

Different physical and chemical parameters including moisture content, specific gravity, color, odor, acid value, viscosity, oxidative stability, triglyceride content, peroxide value, anisidine value, iodine value and free fatty acids, among others, are used to assess the compositional quality of vegetable oils [7,28,29]. These quality assessments are usually done with conventional techniques such as pycnometry, titration and standard AOAC methods [7,30]. Modern spectroscopic techniques including near-infrared (NIR), mid-infrared (MIR), Fourier transform infrared (FTIR), Raman spectroscopy (RS), nuclear magnetic resonance (NMR) and hyperspectral imaging (HIS) are used to assess the quality of vegetable oils, but they are beyond the scope of this study [7,31]. This study, however, considered the titration/volumetric technique and standard AOAC methods to determine the chemical properties, namely, the acid value (AV), free fatty acids (FFAs) and peroxide value (PV) of the extracted oil samples. The AV measures the degree of oil spoilage in terms of FFAs from enzymatic activity [2,32]. The higher the AV, the higher the level of FFAs, which translates into decreased oil quality [33]. FFAs are the result of glycerin decomposition in oils [2]. High levels of FFAs cause rancidity as well as changes in the taste and color of the oil [34,35]. It is reported that during the oil refining process, FFAs are neutralized to reduce their undesirable effects/flavor [3,36]. Adeyanju et al. [34] and Cammerer and Kroh [37] published that high temperature, moisture content and presence of lipase are responsible for the formation of FFAs in fat-containing raw materials or oils. The PV measures the degree of either the occurrence of peroxidation or adulteration, which is used to evaluate the quality and stability of oils during storage [2,38,39]. A high peroxide value is an indicator of oxidation level, and the greater the peroxide value, the more oxidized the oil [40]. Oils with peroxide values higher than 9 meq O_2_/kg cause undesirable health problems by increasing reactive oxygen species as well as secondary products of lipid peroxidation that stimulate cardiovascular and inflammatory diseases [41,42]. Maximum levels of these chemical properties have been published in the Codex Alimentarius [36,43].

Generally, adequate information is still needed in a uniaxial process to understand the complexities associated with the mechanical screw pressing and for improving its efficiency as well as the quality of the oil output. Therefore, this study aimed to: determine the maximum oil yield from the bulk oilseeds and the residual oil content in the press cake through continuous loading, determine the physicochemical properties of the extracted oil samples using standard methods and describe the absorbance and transmittance spectra in the wavelength range of 325–600 nm using a UV–VIS spectrophotometer.

## 2. Materials and Methods

### 2.1. Samples and Determination of Moisture Content

**Figure 1:**
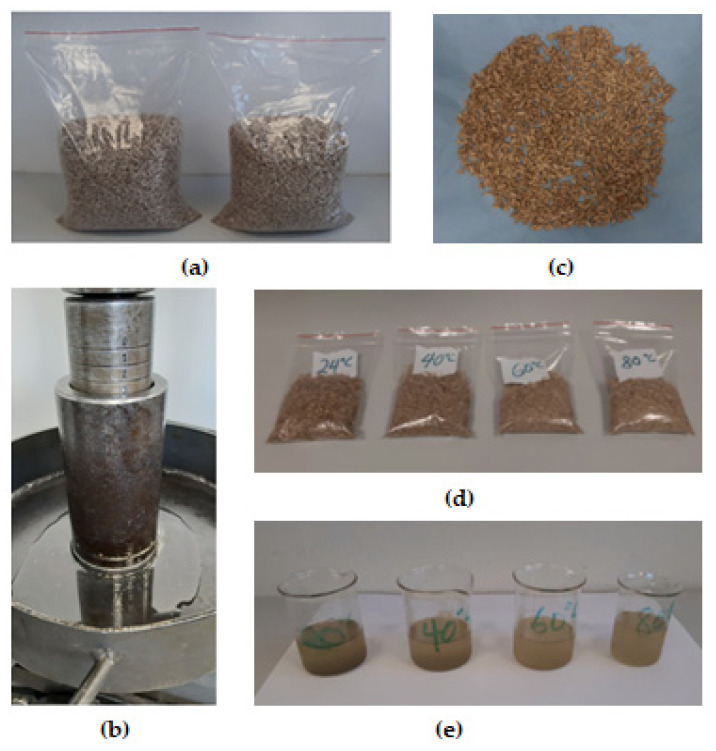
(**a**) Hulled sunflower seeds in sealed transparent plastic bags; (**b**) measured sample loaded in a pressing vessel with a plunger placed atop showing the extracted oil in a metal pan; (**c**) sample after first pressing spread out on a tissue; (**d**) samples after the fifth repeated pressing for each temperature to obtain the residual oil in the seedcake after the first pressing; (**e**) extracted oil in a beaker at each temperature of 24 °C (control), 40 °C, 60 °C and 80 °C.

Eight packets of cleaned hulled sunflower seeds (500 g each making a total of 4 kg) were procured from a supermarket in Prague, Czech Republic. The samples were sealed in two transparent plastic bags (Figure 1a) and kept at a laboratory temperature of 24 °C and humidity of 25%. The initial moisture content of the samples was determined using the conventional oven procedure at a temperature of 105 °C for 24 h [44,45,46]. The tests were conducted twice, and the averaged moisture content value of 4.48 ± 0.19 (% w.b.) was calculated [45]. The electronic balance Kern 440-35 (Kern & Sohn GmbH, Balingen, Germany) with an accuracy of 0.01 g was used for weighing the samples.

### 2.2. Determination of Sample Oil Content

The oil content of hulled sunflower seeds sample was determined by the Soxhlet extraction method [47,48,49]. Approximately 11 g of the sample weight was used. The tests were conducted twice, and the oil content value of 56.09 ± 2.67 (%) was obtained [45].

### 2.3. Samples Pretreatment

The oven (MEMMERT GmbH + Co. KG, Buechenbach, Germany) was used for the pretreatment of the samples at temperatures of 40, 60 and 80 °C before the compression tests to extract the oil. The temperature of 24 °C served as the control for the unheated sample.

### 2.4. Oil Extraction under Uniaxial Process

**Figure 2:**
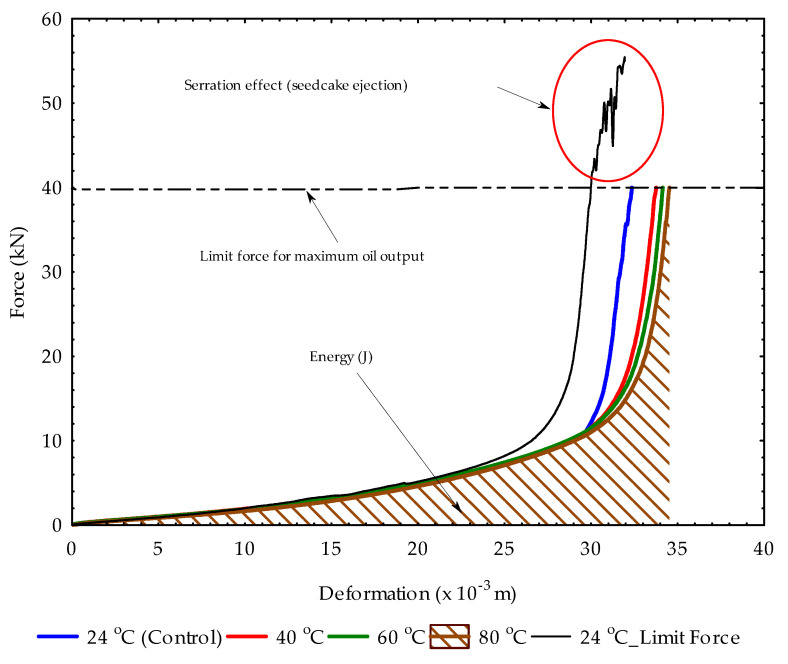
Force and deformation curves of hulled sunflower seeds samples’ oil extraction at different heating temperatures showing the maximum limit force and the serration effect.

Universal compression testing equipment (TEMPOS spol. s.r.o., Opava, Czech Republic (Machine Service), a ZDM 50, VEB Werkstoffprüfmaschinen Leipzig, Germany) and a pressing vessel of 60 mm diameter with a plunger were used to extract the oil from the hulled sunflower seed samples at a control temperature of 24 °C and heating temperatures of 40, 60 and 80 °C at a preset load of 40 kN (equivalent pressure of 14.147 MPa), speed of 4 mm/min and sample pressing height of 60 mm (sample volume of 16.965 × 10^–5^ m^3^ or an initial weight of 103.29 g to a final weight of 65.31 ± 0.70 g) (Figure 1b–e). The preset load (limit force) was determined from a preliminary test, being the curve with the serration effect, which was characterized by the ejection of the seedcake through the bottom holes of the pressing vessel (Figure 2). After the first pressing at 60 mm to obtain the maximum oil from the bulk seed samples, a repeated pressing was done to recover the residual oil in the seedcake until there was no need for further pressings (plastic deformation). The second, third, fourth and fifth repeated pressings were done at pressing heights of 50 mm, 40 mm, 35 mm and 30 mm, respectively. In all, at each temperature, five separate tests were conducted, making a total of 40 tests and repeated twice. From the compression tests and the data obtained, the oil yield, oil expression efficiency and deformation energy were calculated according to the following equations (Equations (1)–(3)) [25,50,51,52]:

(1) $$O_{YD}=\left[\left(\frac{m_{OL}}{m_{SD}}\right)\;100\right]$$

where $O_{YD}$ is the oil yield (%), and $m_{OL}$ is the mass of oil obtained as the difference of the mass of the seed cake and the initial mass of the sample $m_{SD}$ (g).

(2) $$O_{EF}=\left[\left(\frac{O_{YD}}{O_{LC}}\right)\;100\right]$$

where $O_{EF}$ is the oil expression efficiency (%), and $O_{LC}$ is the percentage sample oil content (%) determined by the Soxhlet extraction method.

(3) $$E_{NG}=\overset{n=i-1}{\underset{n=0}{\sum }}\left[\left(\frac{F_{n+1}+F_{n}}{2}\right)\;\left(x_{n+1}-x_{n}\right)\right]$$

where $E_{NG}$ is the deformation energy (J) characterized by the area under the force–deformation curve based on the trapezoidal rule, $F_{n+1}+F_{n}$ and $x_{n+1}-x_{n}$ are the compressive force (kN) and deformation (mm), *n* is the number of data points and *i* is the number of sections in which the axis deformation was divided. The deformation values were directly obtained from the compression data. The sample volume was calculated using Equation (4) [22]:

(4) $$V_{SP}=\left[\left(\frac{\pi \;D^{2}}{4}\right)\;PH\right]$$

where $V_{SP}$ is the sample volume (m^3^), D is the pressing vessel diameter (mm) and PH is the sample pressing height (mm).

### 2.5. Determination of Oils Quality Parameters

The quality parameters, namely, the peroxide value, PV (meq O_2_/kg oil), the acid value, AV (mg KOH/g oil), and the free fatty acid, FFA (mg KOH/g oil), of the extracted oil samples both unheated at 24 °C and heated at 40, 60 and 80 °C were determined according to the published procedures and reagents [2,49,53].

### 2.6. Measurement of UV Spectral Parameters

The extracted oil samples at different temperatures were analyzed for absorbance and transmittance values at a wavelength in the range of 325–600 nm using a UV–VIS spectrophotometer (SpektrofotometrOnda VIS V-10 Plus, Giorgio Bormac S.r.l., Carpi, Italy). This analysis was done following the study conducted by Kumar and Viswanathan [12] and Orozco et al. [54] on UV transmission of edible oils, chicken oil and biodiesel. Distilled water was used as a reference or control for the absorbance and transmittance measurements.

### 2.7. Statistical Analyses

The data were subjected to statistical analyses using STATISTICA 13 software [55] by applying basic statistics (correlation analysis), general linear models (repeated measures ANOVA and multiple regression) and Duncan post hoc tests at a 5% significance level.

## 3. Results

### 3.1. Compression Curves at Initial and Repeated Pressings

**Figure 3:**
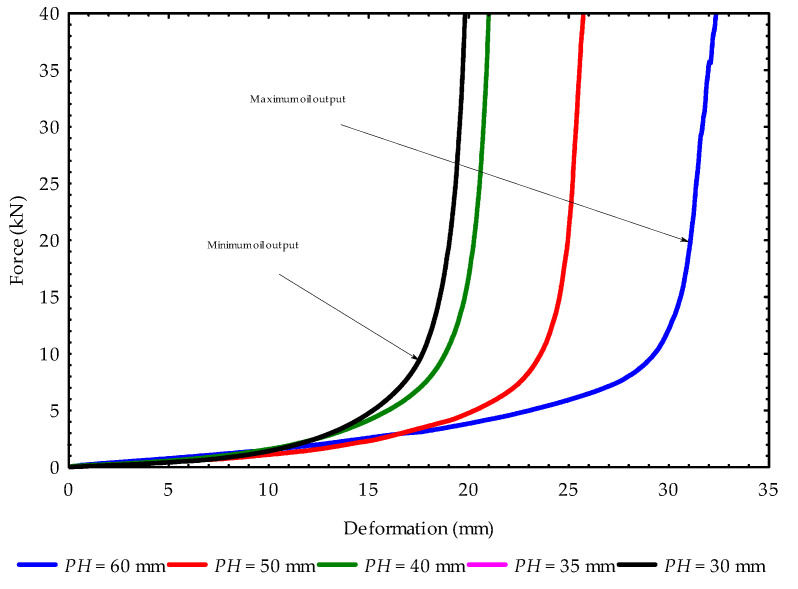
Force–deformation curves of hulled sunflower seed oil extraction at repeated pressings for the control temperature of 24 °C (PH = 60 mm is the initial pressing height) represent the temperatures of 40, 60 and 80 °C for maximum oil output. The pink curve is under the black curve.

The force–deformation curves of the initial and repeated pressings of the hulled sunflower seeds samples are presented in Figure 3. The first or initial pressing was at a height of 60 mm, which produced a maximum oil output (blue curve) (Figure 3). The pressing heights from 50 mm (second pressing) to 30 mm (fifth pressing) decreased in oil output, or a minimum oil amount was recorded (black and pink curves) (Figure 3). The repeated compression was done mainly to recover the residual oil in the samples after the first pressing. At the fifth pressing height of 30 mm, there was no need for further pressing, since almost a plastic deformation of the samples had occurred with a minimum oil flow.

### 3.2. Calculated Parameters and Statistical Evaluation

**Figure 4:**
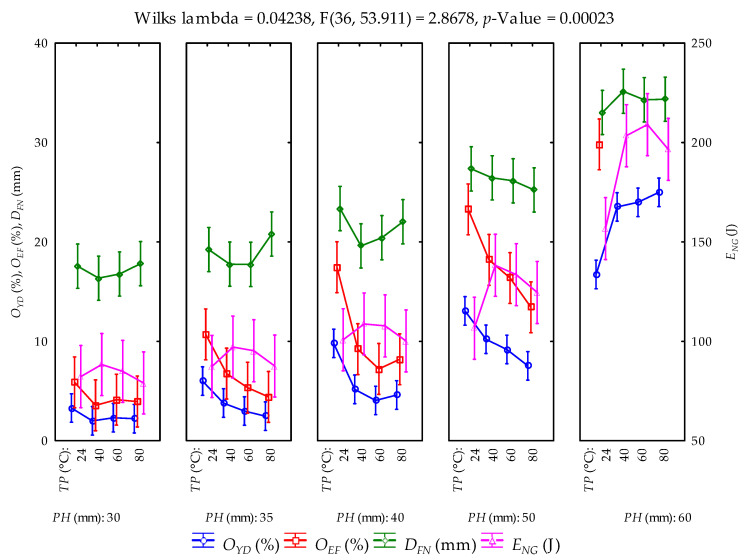
Least square means of the calculated parameters and their factors’ interactions (vertical bars denote 95% confidence interval).

**Figure 5:**
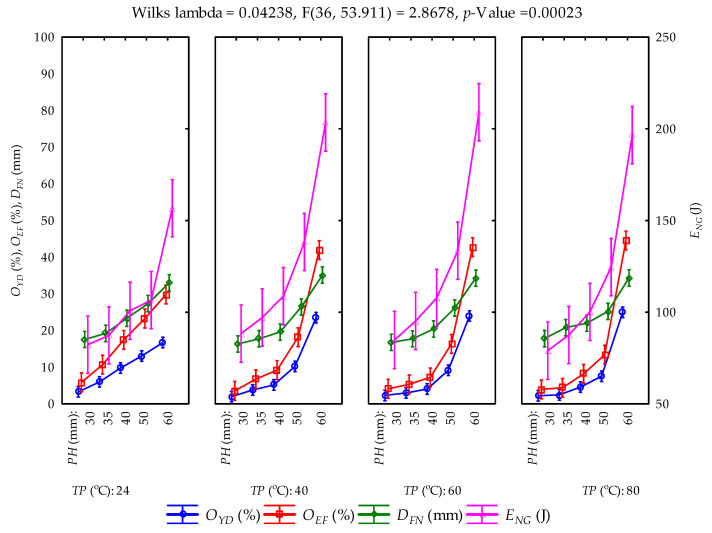
Least square means of the calculated parameters and their factors’ interactions (vertical bars denote 95% confidence interval; *PH*: pressing height; *TP*: temperature; $O_{YD}$: oil yield; $O_{EF}$: oil expression efficiency; $D_{FN}$: deformation; $E_{NG}$: energy).

**Figure 6:**
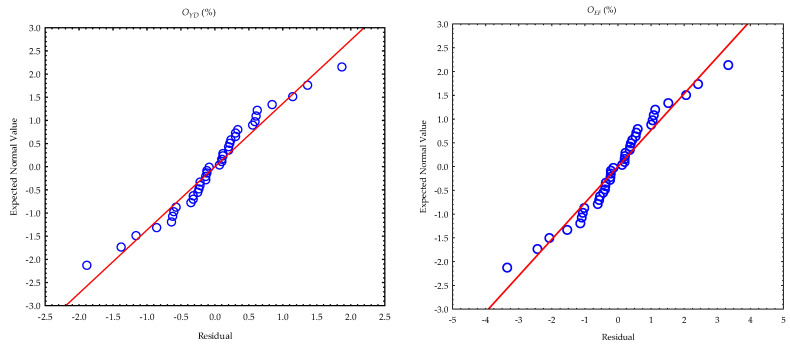

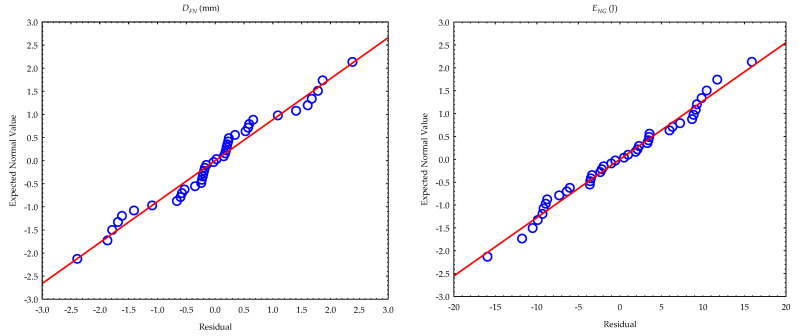
Normal probability plots of the residuals of the calculated parameters ($O_{YD}$: oil yield; $O_{EF}$: oil expression efficiency; $D_{FN}$: deformation; $E_{NG}$: energy).

The means and standard deviations of the calculated parameters (oil yield, oil expression efficiency, deformation and deformation energy (hereinafter referred to as energy)) from the compression tests at the various pressing heights and temperatures are provided in Table 1. The samples were repeatedly pressed to obtain the maximum oil output from the bulk oilseeds and the residual oil in the press/seedcake. The total amounts of oil yield ranged from 48.869 ± 6.023% to 41.798 ± 3.557%. Oil expression efficiency ranged from 87.117 ± 10.736% to 74.512 ± 6.341%. Deformation and energy values ranged from 120.510 ± 7.679 mm to 120.210 ± 3.748 mm and from 533.709 ± 65.644 J to 587.932 ± 15.183 J, respectively. At an initial pressing height of 60 mm, oil yield and oil expression efficiency showed an increment with the increase in heating temperatures, whereas deformation values decreased but energy values increased from temperatures of 24 °C until 60 °C and then decreased at 80 °C (Figure 4). Regarding the repeated samples at the pressing heights between 50 mm and 30 mm, oil yield, oil expression efficiency and energy decreased with temperature in contrast to deformation values, which increased at the pressing heights of 40 mm, 35 mm and 30 mm. Generally, all the calculated parameters decreased along with the pressing heights from 60 mm through to 30 mm for all the heating temperatures between 40 °C and 80 °C and the control temperature of 24 °C (Figure 5). The multivariate tests of significance and test of the whole model of the effect of the factors and their interactions on the calculated parameters are provided in Table 2 and Table 3. The significance of the tests was established based on the fact that the *p*-value was less than the 5% probability level. This implies that the varying factors (pressing height and temperature) significantly influenced the calculated parameters. The coefficient of determination (R^2^) values ranged from 0.972 to 0.992, respectively. The principal parameters (oil yield and energy) were further subjected to post hoc tests by employing the Duncan test of homogeneity (Table 4 and Table 5). By arranging the means of the principal parameters from the lowest to the highest, twelve groups of homogeneous means represented as four stars (****) were observed for oil yield, where seven of those were observed for energy. The means of the same group indicate no significant difference from each other, whereas the different groups underscore significant differences from each. The normal probability plots of the calculated parameters are illustrated in Figure 6. The normality assumption was satisfied since the data points approximately followed a straight line.

### 3.3. Quality Indicators of Oils and Statistical Evaluation

The moisture content of the hulled sunflower seeds samples was found to be 4.48 ± 0.19 (% w.b.). The extracted oil samples at a control temperature of 24 °C (unheated) and heated at temperatures of 40, 60 and 80 °C were evaluated in terms of peroxide value, PV (meq O_2_/kg oil), acid value, AV (mg KOH/g oil) and free fatty acid, FFA (mg KOH/g oil) (Table 6). The PV values ranged from 6.898 ± 0.144 to 7.290 ± 0.507, the AV values ranged from 1.043 ± 0.166 to 1.998 ± 0.276, and the FFA values ranged from 0.521 ±0.083 to 0.999 ± 0.138. The one-way ANOVA analysis showed that the heating temperatures in comparison with the control temperature had no significant effect (*p* < 0.05) on the quality properties of the oil samples. The coefficients of determination values ranged from 0.242 to 0.766 (Table 7).

### 3.4. UV Spectral Properties of Oils

**Figure 7:**
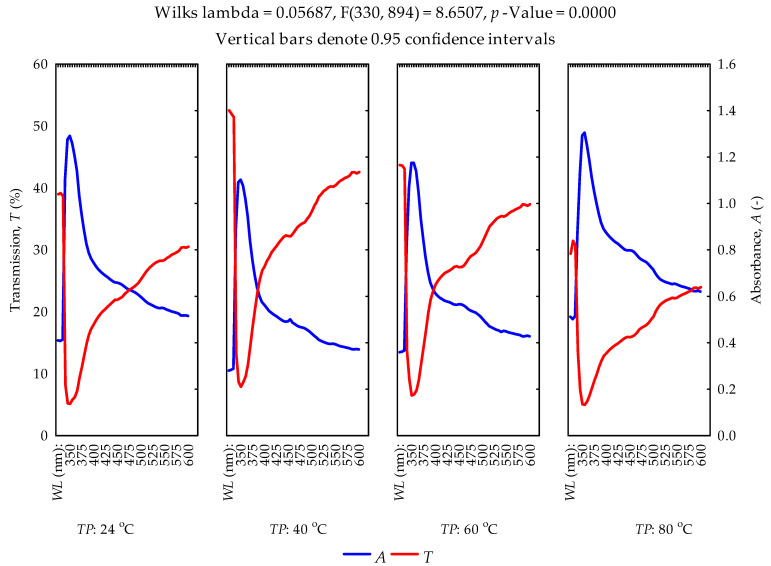
Transmittance and absorbance versus wavelength (nm) curves at various pretreatment temperatures (24 °C—unheated as the control).

The data on the absorption and transmittance spectra within the wavelength range of 325–600 nm are described in Supplementary Materials (Section 3.5). The results of the multiple regression and multivariate tests of significance are presented (Table 8, Table 9 and Table 10). The coefficients of the regressors (wavelength and temperature), as well as the intercept, were significant (*p* < 0.05) for predicting the absorption rate. Regarding the model for the transmittance rate, the coefficient of the intercept was not significant (*p* > 0.05), while the other regressors proved significant (*p* < 0.05). The values of the coefficient of determination were 0.351 and 0.298, respectively. Based on the multivariate tests of significance, the interactions of the wavelength and temperature had a significant effect (*p* > 0.05) on the absorption and transmittance rates of the hulled sunflower seeds oil samples. Strong absorption peaks of the oil samples for the various temperatures were noticed at wavelength values between 350 and 360 nm. However, the absorption rates decreased from 1.305 ± 0.035 to 0.280 ± 0.026 in the wavelength range of 365–600 nm. The transmittance values ranged from 5.200 ± 0.346 to 52.533 ± 3.002% (Figure 7).

### 3.5. Supplementary Materials

The descriptive statistics (means, standard deviations and standard errors) of the effect of heating temperatures (40, 60 and 80 °C) on the absorbance and transmittance rates in the wavelength range from 325 to 600 nm are provided (Table S1). The scatterplots of the absorbance and transmittance versus wavelength at a specific heating temperature correlated both negatively (r = −0.558) and positively (r = 0.494) (Figures S1 and S2). On the contrary, the scatterplots of the absorbance and transmittance versus temperature at a specific wavelength correlated positively (r = 0.198) and negatively (r = −0.232) (Figures S3 and S4). It can be stated that the heating temperatures had a significant effect on the absorbance and transmittance rates of hulled sunflower seeds oil samples.

## 4. Discussion

The purpose of this study was to estimate the maximum oil recovery efficiency and residual oil from hulled sunflower bulk seeds and press cakes at various temperatures (24 °C, 40 °C, 60 °C and 80 °C) in the uniaxial compression process. The corresponding energies were calculated. In addition, the extracted oil samples were evaluated in terms of physicochemical properties (moisture content, peroxide value, acid value and free fatty acid) and UV spectral curves (absorbance and transmittance in the wavelength range of 325–600 nm). To extract the oil, the maximum load of 40 kN corresponding to a pressure of 14.147 MPa (ratio of force to the area of pressing vessel) was combined with a pressing speed of 4 mm/min for an initial sample pressing height of 60 mm using the vessel diameter of 60 mm. Here the sample volume of 16.965 × 10^–5^ m^3^ was calculated (Equation (4)). The maximum preset load was determined from a preliminary test, where the force–deformation curve at 60 kN showed a serration pattern characterized by the ejection of the seedcake through the bottom holes of the pressing vessel. According to Divisova et al. [22], the maximum oil yield from oilseed samples is attained under the curve without the serration effect. Based on the compression data obtained, the parameters, namely, oil yield, oil expression efficiency and energy, were calculated with the varied pressings at heights of 60 mm to 30 mm (samples volume in the range of 14.137 × 10^–5^ m^3^ to 8.482 × 10^–5^ m^3^); heating temperatures of 40, 60 and 80 °C; and a control temperature of 24 °C (unheated sample).

For samples at an initial pressing height of 60 mm against temperature, the oil yield and/or oil expression efficiency increased along with the increase in heating temperatures. An energy increment was realized for heating temperatures between 24 °C and 60 °C but declined at 80 °C. This meant that at a heating temperature of 80 °C, a higher oil recovery efficiency of 44.545 ± 1.489% was found with a lower energy demand of 196.548 ± 1.426 J in comparison with a heating temperature of 60 °C, which gave a lower oil recovery efficiency of 42.759 ± 0.305% but with a higher energy requirement of 209.032 ± 13.975 J. With repeated pressings at heights of 50 mm to 30 mm with the temperatures, all the determined parameters decreased in amounts because of the first pressing, which was initiated at 60 mm of height. However, it was observed that without the repeated pressing from the first pressing, a considerable amount of oil would have remained in the press/seedcake. Here, the total residual oil yield amounts obtained at all temperatures were 32.145 ± 5.673%, 21.168 ± 2.551%, 18.537 ± 1.013% and 16.810 ± 2.722%, respectively. The corresponding energy values were 377.012 ± 60.596 J, 432.458 ± 29.606 J, 421.403 ± 36.647 J and 391.385 ± 13.757 J. This result indicates that more residual oil with a minimum energy input is thus obtained from the sample temperature of 24 °C without the heat treatment compared to the samples with heat treatment. This agreed with the cumulative amounts of all the pressings carried out (initial pressing combined with the repeated pressings). Nevertheless, a higher energy demand was required to recover the residual oil in the press cake than the maximum oil yield obtained from the bulk oilseeds at the initial/first pressing of 60 mm for the varied temperatures (Table 1). The present results agree with the findings of Karaj and Muller [20], Khan and Hanna [56] and Li et al. [57], cited in Deng et al. [58], who indicated that lower energy input results in lower oil recovery efficiency, leading to higher oil residue in press cake and higher seed material throughput; oil yield of cottonseeds increased from 13.0 to 24.2% as the temperature increased from 18 °C to 125 °C, and oil yield for peony seeds increased at a temperature of 73 °C and moisture content of 4.6% under a pressure of 4.6 MPa and feed rate of 1600 g/min.

For the physicochemical properties of the extracted oil samples, the moisture content is an indirect measure of the oil’s quality and resistance to thermal oxidation [59]. Hoffmann [60] and Reuber [61] suggested that lower moisture content of oilseeds increases friction, whereas higher moisture content acts as a lubricant. Although higher oil recovery could be achieved at a lower moisture content, in the case of screw pressing it is not advisable to press oilseeds below a moisture content of 3.6% due to plugging [62]. Heat pretreatment of oilseeds is usually done to induce higher oil yield, which thus affects the oil quality [62]. In this study, the oil quality parameters, namely, the peroxide value (PV), acid value (AV) and free fatty acid (FFA) were determined at different heating temperatures (40 °C, 60 °C and 80 °C) and compared to the control temperature of 24 °C. The peroxide value increased from 6.898 ± 0.144 to 7.290 ± 0.507 meq O_2_/kg at temperatures of 24 °C to 60 °C but decreased at 80 °C. The acid value and free fatty acid (mg KOH/g oil) increased up to 40 °C but decreased at 60 °C and then increased at 80 °C. However, the increment at 80 °C was lower than at 40 °C and higher than at 24 °C (Table 6). The extracted oil samples were not significantly (*p* > 0.05) affected by the heating temperatures. Choo et al. [63], cited in Herchi et al. [29], reported that during heating there is an accumulation of peroxides. Konuskan et al. [42] mentioned that high temperature, visible light and oxygen can easily increase the peroxide value of the oils. Herchi et al. [29] also indicated PV, AV and FFA values of hull flaxseed oil extracted at 110 °C for 12 h. The authors published PV values between 1.85 ± 0.08 and 5.2 ± 0.15 meq O_2_/kg oil, the AV values between 1.5 ± 0.14 and 2.9 ± 0.25 mg KOH/g oil and the FFA values between 0.9 ± 0.06 and 1.7 ± 0.10% oleic acid. The authors further indicated that the increase in PV values showed that the oil was unstable due to oxidative degradation. On the other hand, heating caused an increase in AV, whereas the FFA increase could be attributed to oxidation and hydrolysis that produce FFAs. Adeyanju et al. [34] reported FFA values in the range of 5.34 and 1.86% for coconut oil at temperatures between 60 and 120 °C and roasting time between 5 and 30 min. The authors stated that FFAs decreased with a decrease in roasting temperature and time. Ajai et al. [64] published the above-mentioned properties of selected vegetable oils (groundnut, soybean, palm oil and palm kernel oil) at laboratory temperatures. Their results for PV ranged from 1.90 ± 0.30 to 6.97 ± 0.41 meq O_2_/kg oil and AV ranged from 0.42 ± 0.03 to 1.13 ± 0.21 mg NaOH/g. For refined sunflower oil, 0.13% equivalent in oleic acid has been reported [65]. According to the Codex Alimentarius [44] and Eke-Ejiofor et al. [66], the permissible levels of peroxide values for virgin oils and cold pressed fats and oils is up to 15 milliequivalents of active oxygen/kg oil, and for other fats and oils is up to 10 milliequivalents of active oxygen/kg oil; the acid value for refined fats and oils is 0.6 mg KOH/g fat or oil, for virgin fats and oils is 4.0 mg KOH/g fat or oil and for cold pressed fats and oils is 4.0 mg KOH/g fat or oil, and FFA of 0.6%. FFA is half the AV [67].

The absorbance and transmittance curves of the extracted oil samples at different temperatures within the wavelength range of 325–600 nm were described (Figure 7). The increase in heating temperature increased the absorption rate. The refraction/inverse of the absorbance is the transmittance or vice versa [68]. In the study by Kumar and Viswanathan [12], the authors reported UV absorption and transmittance spectra of selected vegetable oils, namely, mustard oil, sesame oil, neem oil, coconut oil, castor oil and groundnut oil, as well as cod liver oil and chicken oil in the wavelength range of 200–400 nm. The authors indicated that most of the oils showed poor absorption in the UVB region (280–320 nm), while others showed a moderate absorption rate, but chicken oil showed high absorption. In addition, the authors observed transmission rates between 20 and 100% in all the oils they studied. This study, however, observed an absorption peak at 350 nm for all temperatures, which suggests that the oils may contain the natural pigment molecules chlorophyll and carotenoid, belonging to porphyrins and terpenoids [69,70]. Ref. [70] further indicated that β carotene and chlorophyll were the major factors that caused the difference in absorption spectra. The absorption and transmission rates were between 0.28 and 1.305 (-) and 5 and 52.53%, respectively. The differences in comparison with the above-mentioned study [12] could be due to the oil type used and the heating pretreatment. The experimental data (means, standard deviations and standard errors) are provided in the Supplementary Material. Statistically, the standard deviation is a measure of spread and variability, whereas the standard error is a measure of the precision of the sample mean [71]. The more spread out the data distribution, the higher the standard deviation and vice versa. The smaller values of the standard deviation and standard error show the reliability of the mean and thus explain the normal distribution of the data at a 95% confidence interval.

## 5. Conclusions

Samples of hulled bulk sunflower seeds subjected to various heating temperatures of 40 °C, 60 °C and 80 °C together with an unheated sample temperature of 24 °C were examined for high percentage oil yield and/or oil expression efficiency with a minimum energy requirement under a uniaxial compression process using a pressing vessel of diameter 60 mm under a load of 40 kN and speed of 4 mm/min. After the first/initial pressing at a height of 60 mm (sample volume of 16.965 × 10^–5^ m^3^); repeated pressings at heights from 50 mm to 30 mm (sample volume in the range of 14.137 × 10^–5^ m^3^ to 8.482 × 10^–5^ m^3^) were done to recover the residual oil remaining in the press/seedcake by applying the same load and speed for each pressing. The first pressing produced a minimum oil yield, leaving a considerable amount of the residual oil in the sample press cake. Heating the samples enhanced the oil recovery; however, maximum oil yield was obtained at the fifth repeated pressing. Cumulatively, oil yields of 48.869 ± 1.466%, 44.689 ± 1.296%, 42.523 ± 0.510% and 41.798 ± 1.887% were obtained for the heating temperatures and the control temperature, respectively. The heating temperatures compared to the control temperature had no significant effect (*p* < 0.05) on the chemical properties of the oil samples (peroxide value, acid value and free fatty acid), which were within the recommended limit for edibility. A single absorption peak was observed at 350 nm for all the oil samples indicating low levels of pigment molecules (chlorophyll and carotenoids) in the oil. The absorption and transmission rates were, respectively, between 0.280 ± 0.026 and 1.305 ± 0.035 and from 5.200 ± 0.346 to 52.533 ± 3.002%, indicating the possible use of sunflower oil as a skin softener against ultraviolet radiation. Future studies could examine unhulled bulk sunflower seeds and other bulk oilseeds to obtain adequate information on the uniaxial oil extraction process towards improving the mechanical screw pressing operation. In addition, appropriate analytical tools such as FT-IR spectroscopy combined with multivariate statistical techniques could be employed to characterize the oil samples concerning the pretreatment conditions.

## Figures and Tables

**Table 1:** Calculated parameters from the repeated pressings of hulled sunflower seeds samples.

Pressing Height, PH (mm)	Temperature, TP (°C)	Oil Yield, $O_{YD}$ (%)	Oil Expression Efficiency, $O_{EF}$ (%)	Deformation, $D_{FN}$ (mm)	Energy, $E_{NG}$ (J)
60 ^a^	24 *	16.725 ± 0.349	29.815 ± 0.622	33.020 ± 0.933	156.698 ± 5.048
50 ^b^		13.060 ± 1.929	23.281 ± 3.439	27.350 ± 2.277	106.598 ± 14.802
40 ^b^		9.791 ± 0.801	17.454 ± 1.427	23.355 ± 3.373	100.788 ± 22.546
35 ^b^		6.003 ± 2.652	10.701 ± 4.727	19.225 ± 0.813	87.367 ± 12.951
30 ^b^		3.291 ± 0.292	5.866 ± 0.520	17.560 ± 0.283	82.260 ± 10.296
Sum ± SD		48.869 ± 6.023	87.117 ± 10.736	120.510 ± 7.679	533.709 ± 65.644
60 ^a^	40	23.521 ± 0.897	41.930 ± 1.599	35.155 ± 1.987	203.426 ± 16.646
50 ^b^		10.206 ± 1.635	18.194 ± 2.914	26.445 ± 0.318	138.270 ± 13.150
40 ^b^		5.169 ± 0.442	9.214 ± 0.788	19.600 ± 0.212	108.731 ± 9.006
35 ^b^		3.793 ± 0.295	6.761 ± 0.525	17.765 ± 2.638	97.129 ± 4.719
30 ^b^		2.000 ± 0.179	3.565 ± 0.319	16.355 ± 2.524	88.329 ± 2.730
Sum ± SD		44.689 ± 3.448	79.665 ± 6.146	115.320 ± 7.769	635.883 ± 46.252
60 ^a^	60	23.986 ± 0.171	42.759 ± 0.305	34.305 ± 0.304	209.032 ± 13.975
50 ^b^		9.183 ± 0.105	16.370 ± 0.188	26.150 ± 0.269	133.532 ± 12.344
40 ^b^		4.053 ± 0.432	7.225 ± 0.770	20.425 ± 0.488	107.773 ± 12.566
35 ^b^		2.990 ± 0.310	5.330 ± 0.552	17.740 ± 1.541	95.251 ± 3.264
30 ^b^		2.312 ± 0.166	4.122 ± 0.296	16.775 ± 0.841	84.848 ± 8.473
Sum ± SD		42.523 ± 1.184	75.805 ± 2.111	115.395 ± 3.444	630.435 ± 50.621
60 ^a^	80	24.988 ± 0.835	44.545 ± 1.489	34.355 ± 0.247	196.548 ± 1.426
50 ^b^		7.530 ± 1.211	13.424 ± 2.159	25.230 ± 2.376	124.590 ± 0.744
40 ^b^		4.599 ± 0.866	8.198 ± 1.543	22.025 ± 0.035	100.181 ± 3.053
35 ^b^		2.470 ± 0.166	4.402 ± 0.296	20.790 ± 0.339	87.584 ± 4.897
30 ^b^		2.212 ± 0.479	3.943 ± 0.853	17.810 ± 0.750	79.030 ± 5.062
Sum ± SD		41.798 ± 3.557	74.512 ± 6.341	120.210 ± 3.748	587.932 ± 15.183

^a^ Initial pressing; ^b^ repeated pressing to obtain the residual/maximum oil; * control at laboratory temperature; SD: standard deviation.

**Table 2:** Multivariate tests of significance of the factors’ effects on the calculated parameters.

Effect	Test	Value	*F*-Value	Effect df	Error df	*p*-Value
Intercept	Wilks lambda	0.001	4413.75	3	18	<0.05
Pressing height, *PH* (mm)	Wilks lambda	0.003	30.97	12	47.915	<0.05
Temperature, *TP* (°C)	Wilks lambda	0.229	4.064	9	43.958	<0.05
*PH* × *TP*	Wilks lambda	0.042	2.868	36	53.911	<0.05

df: degrees of freedom; *p*-value < 0.05 implies significance.

**Table 3:** Whole model test for the effect of the factors on the calculated parameters.

Calculated Parameters	Multiple R	Multiple R^2^	Adjusted R^2^	F-Value	*p*-Value
$O_{YD}$ (%)	0.996	0.992	0.984	124.332	<0.05
$O_{EF}$ (%)	0.996	0.992	0.984	124.332	<0.05
$D_{FN}$ (mm)	0.986	0.972	0.945	35.974	<0.05
$E_{NG}$ (J)	0.983	0.967	0.935	30.524	<0.05

R: correlation; R^2^: coefficient of determination; $O_{YD}$: oil yield; $O_{EF}$: oil expression efficiency; $D_{FN}$: deformation; $E_{NG}$: energy.

**Table 4:** Duncan test of homogenous means of the calculated parameter $O_{YD}$: oil yield (%) for *PH* and *TP* interactions.

Cell No.	*PH* (mm)	*TP* (°C)	$O_{YD}$ (%) * Means	1	2	3	4	5	6	7	8	9	10
2	30	40	2.000	****									
4	30	80	2.212	****									
3	30	60	2.312	****	****								
8	35	80	2.470	****	****								
7	35	60	2.990	****	****	****							
1	30	24	3.291	****	****	****							
6	35	40	3.793	****	****	****	****						
11	40	60	4.053	****	****	****	****						
12	40	80	4.599		****	****	****						
10	40	40	5.169			****	****						
5	35	24	6.003				****	****					
16	50	80	7.530					****	****				
15	50	60	9.183						****	****			
9	40	24	9.791							****			
14	50	40	10.206							****			
13	50	24	13.060								****		
17	60	24	16.725									****	
18	60	40	23.521										****
19	60	60	23.986										****
20	60	80	24.988										****

*PH*: pressing height; *TP*: temperature; * means arranged from the lowest to the highest; **** homogeneous means/groups of means at 0.05 significance level.

**Table 5:** Duncan test of homogenous means of the calculated parameter $E_{NG}$: energy (J) for *PH* and *TP* interactions.

Cell No.	*PH* (mm)	*TP* (°C)	$E_{NG}$ (J) * Means	1	2	3	4	5	6	7
4	30	80	79.030	****						
1	30	24	82.260	****	****					
3	30	60	84.848	****	****	****				
5	35	24	87.367	****	****	****				
8	35	80	87.584	****	****	****				
2	30	40	88.329	****	****	****				
7	35	60	95.251	****	****	****				
6	35	40	97.129	****	****	****				
12	40	80	100.181	****	****	****	****			
9	40	24	100.788	****	****	****	****			
13	50	24	106.598		****	****	****			
11	40	60	107.773		****	****	****			
10	40	40	108.731			****	****			
16	50	80	124.590				****	****		
15	50	60	133.532					****		
14	50	40	138.270					****	****	
17	60	24	156.698						****	
20	60	80	196.548							****
18	60	40	203.426							****
19	60	60	209.032							****

*PH*: pressing height; *TP*: temperature; * means arranged from the lowest to the highest; **** homogeneous means/groups of means at 0.05 significance level.

**Table 6:** Mean and standard deviation of the chemical properties of the extracted oil samples.

Temperature (°C)	Peroxide Value, PV (meq O_2_/kg Oil)	Acid Value, AV (mg KOH/g Oil)	Free Fatty Acid, FFA (mg KOH/g Oil)
24 *	6.898 ± 0.144	1.567 ± 0.350	0.783 ± 0.175
40	7.068 ± 0.403	1.998 ± 0.276	0.999 ± 0.138
60	7.290 ± 0.507	1.043 ± 0.166	0.521 ± 0.083
80	6.957 ± 0.347	1.698 ± 0.257	0.849 ± 0.128

* Control at laboratory temperature.

**Table 7:** One-way ANOVA analysis of the chemical properties of the extracted oil samples.

Chemical Properties	R^2^	*F*-Value	*p*-Value
Peroxide value, PV (meq O_2_/kg)	0.242	0.426	>0.05
Acid value, AV (mg KOH/g oil)	0.765	4.351	>0.05
Free fatty acid, FFA (mg KOH/g oil)	0.766	4.358	>0.05

R^2^: coefficient of determination; *p*-value > 0.05 implies non-significance.

**Table 8:** Absorbance parameter estimates and statistical evaluation.

Factors Effect	Parameter, A (-) ^a^	Sum of Squares	Mean Squares	*F*-Value	*p*-Value
Intercept	1.26584	27.176	27.176	801.688	<0.05
*WL*	−0.00156	10.890	10.890	321.260	<0.05
*TP*	0.00214	1.369	1.369	40.379	<0.05

*WL*: wavelength (nm); *TP*: temperature (°C); *A*: absorbance; ^a^ coefficient of determination, R^2^ = 0.351; *p*-value < 0.05 implies significance.

**Table 9:** Transmittance parameter estimates and statistical evaluation.

Factors Effect	Parameter, *T* (%) ^a^	Sum of Squares	Mean Squares	*F*-Value	*p*-Value
Intercept	1.34048	30.48	30.48	0.3858	>0.05
*WL*	0.06468	18,362.42	18,362.42	232.4536	<0.05
*TP*	−0.11678	4059.91	4059.91	51.3952	<0.05

*WL*: wavelength (nm); *TP*: temperature (°C); *T*: transmittance; ^a^ coefficient of determination, R^2^ = 0.298; *p*-value > 0.05 implies non-significance; *p*-value < 0.05 implies significance.

**Table 10:** Multivariate tests of significance of the factors’ effects on absorbance and transmittance.

Factor Effects	Test	Value	*F*-Value	Effect df	Error df	*p*-Value
Intercept	Wilks lambda	0.000124	1799571	2	447	<0.05
*WL*	Wilks lambda	0.001361	212	110	894	<0.05
*TP*	Wilks lambda	0.040675	590	6	894	<0.05
*WP* × *TP*	Wilks lambda	0.056873	9	330	894	<0.05

*WL*: wavelength (nm); *TP*: temperature (°C); df: degrees of freedom; *p*-value < 0.05 implies significance.

## Data Availability

The data presented in this study are available upon request from the corresponding author.

## Supplementary Materials

The following supporting information can be downloaded at: https://www.mdpi.com/article/10.3390/foods11182866/s1, Table S1: Descriptive statistics of the effect of the factors on absorbance and transmittance, Figure S1: Scatterplot of absorbance versus wavelength at 95% confidence interval, Figure S2: Scatterplot of transmittance versus wavelength at 95% confidence interval, Figure S3: Scatterplot of absorbance versus temperature at 95% confidence interval and Figure S4: Scatterplot of transmittance versus temperature at 95% confidence interval.
