# Supplementary material for: Determination of Maximum Oil Yield, Quality Indicators and Absorbance Spectra of Hulled Sunflower Seeds Oil Extraction under Axial Loading
Source: Foods. 2022 Sep 16;11(18):2866. doi: 10.3390/foods11182866 (PMC9498589; doi:10.3390/foods11182866)
Supplement: Supplementary file 1 [file foods-11-02866-s001.zip › foods-1918651-supplementary.pdf]

foods

MDPI

Article

Determination of Maximum Oil Yield, Quality Indicators and Absorbance Spectra of Hulled Sunflower Seeds Oil Extraction under Axial Loading

Abraham Kabutey*, David Herák, Čestmír Mizera

Department of Mechanical Engineering, Faculty of Engineering, Czech University of Life Sciences Prague, 165 20 Prague, Czech Republic
* Correspondence: kabutey@tf.czu.cz (A.K.); Tel.: +42-02-2438-3180

Table S1. Descriptive statistics of the factors effect on absorbance and transmittance.

Effect	Level of factor, WL (nm)	Level of factor, TP (°C)	N	Mean, A (-)	SD, A (-)	SE, A (-)	Mean, T (%)	SD, T (%)	SE, T (%)
WL x TP	325	24	3	0.410	0.041	0.024	39.000	3.635	2.098
	325	40	3	0.280	0.026	0.015	52.533	3.002	1.733
	325	60	3	0.360	0.023	0.013	43.700	2.254	1.301
	325	80	3	0.512	0.022	0.013	29.400	3.900	2.252
	330	24	3	0.408	0.041	0.024	39.167	3.669	2.118
	330	40	3	0.284	0.024	0.014	52.000	2.955	1.706
	330	60	3	0.362	0.026	0.015	43.600	2.524	1.457
	330	80	3	0.502	0.036	0.021	31.467	2.570	1.484
	335	24	3	0.415	0.042	0.024	38.667	3.790	2.188
	335	40	3	0.289	0.029	0.017	51.433	3.329	1.922
	335	60	3	0.367	0.027	0.015	43.067	2.558	1.477
	335	80	3	0.515	0.038	0.022	30.700	2.663	1.537
	340	24	3	1.104	0.079	0.045	8.167	0.961	0.555
	340	40	3	0.890	0.027	0.016	13.333	0.945	0.546
	340	60	3	0.815	0.008	0.005	13.733	0.379	0.219
	340	80	3	0.867	0.013	0.007	13.633	0.666	0.384
	345	24	3	1.275	0.008	0.005	5.200	0.346	0.200
	345	40	3	1.091	0.067	0.038	8.633	1.436	0.829
	345	60	3	1.070	0.022	0.013	9.000	0.346	0.200
	345	80	3	1.127	0.025	0.014	7.367	0.473	0.273
	350	24	3	1.291	0.055	0.032	5.167	0.611	0.353
	350	40	3	1.102	0.078	0.045	7.900	1.480	0.854
	350	60	3	1.175	0.027	0.016	6.500	0.436	0.252
	350	80	3	1.293	0.028	0.016	5.067	0.321	0.186
	355	24	3	1.260	0.048	0.027	5.800	0.361	0.208
	355	40	3	1.078	0.080	0.046	8.600	1.652	0.954
	355	60	3	1.175	0.026	0.015	6.667	0.416	0.240

	355	80	3	1.305	0.035	0.020	5.000	0.400	0.231
	360	24	3	1.208	0.052	0.030	6.233	0.702	0.406
	360	40	3	1.021	0.079	0.046	9.600	1.646	0.950
	360	60	3	1.140	0.041	0.024	7.300	0.721	0.416
	360	80	3	1.252	0.032	0.019	5.567	0.416	0.240
	365	24	3	1.139	0.054	0.031	7.267	0.902	0.521
	365	40	3	0.943	0.083	0.048	11.533	2.113	1.220
	365	60	3	1.049	0.033	0.019	8.933	0.666	0.384
	365	80	3	1.187	0.028	0.016	6.533	0.404	0.233
	370	24	3	1.032	0.052	0.030	9.333	1.106	0.639
	370	40	3	0.839	0.076	0.044	14.633	2.367	1.367
	370	60	3	0.935	0.035	0.020	11.633	0.929	0.536
	370	80	3	1.112	0.030	0.017	7.700	0.529	0.306
	375	24	3	0.955	0.050	0.029	11.100	1.253	0.723
	375	40	3	0.756	0.077	0.044	17.700	2.951	1.704
	375	60	3	0.847	0.030	0.018	14.200	0.964	0.557
	375	80	3	1.055	0.033	0.019	8.833	0.643	0.371
	380	24	3	0.886	0.048	0.027	13.067	1.401	0.809
	380	40	3	0.691	0.068	0.039	20.533	3.066	1.770
	380	60	3	0.769	0.030	0.017	17.033	1.155	0.667
	380	80	3	1.004	0.033	0.019	9.933	0.737	0.426
	385	24	3	0.827	0.045	0.026	14.933	1.550	0.895
	385	40	3	0.636	0.060	0.035	23.233	3.086	1.782
	385	60	3	0.704	0.030	0.017	19.833	1.332	0.769
	385	80	3	0.956	0.023	0.013	11.100	0.557	0.321
	390	24	3	0.788	0.045	0.026	16.367	1.701	0.982
	390	40	3	0.600	0.053	0.031	25.200	2.955	1.706
	390	60	3	0.659	0.028	0.016	21.967	1.365	0.788
	390	80	3	0.917	0.016	0.009	12.100	0.458	0.265
	395	24	3	0.763	0.045	0.026	17.300	1.808	1.044
	395	40	3	0.576	0.049	0.028	26.667	2.892	1.670
	395	60	3	0.634	0.029	0.017	23.267	1.537	0.888
	395	80	3	0.891	0.016	0.009	12.867	0.513	0.296
	400	24	3	0.746	0.044	0.025	18.000	1.808	1.044
	400	40	3	0.564	0.040	0.023	27.333	2.570	1.484
	400	60	3	0.615	0.029	0.017	24.333	1.607	0.928
	400	80	3	0.877	0.018	0.010	13.267	0.551	0.318
	405	24	3	0.730	0.045	0.026	18.667	1.955	1.129
WL x TP	405	40	3	0.551	0.037	0.022	28.200	2.364	1.365
	405	60	3	0.603	0.030	0.017	24.967	1.629	0.940
	405	80	3	0.864	0.020	0.011	13.700	0.656	0.379
	410	24	3	0.717	0.045	0.026	19.233	2.003	1.157
	410	40	3	0.538	0.029	0.017	28.833	2.255	1.302
	410	60	3	0.594	0.028	0.016	25.467	1.617	0.933
	410	80	3	0.852	0.022	0.013	14.067	0.751	0.433
	415	24	3	0.707	0.046	0.027	19.733	2.103	1.214
	415	40	3	0.529	0.025	0.014	29.600	1.670	0.964

	415	60	3	0.588	0.030	0.017	25.900	1.735	1.002
	415	80	3	0.843	0.023	0.013	14.367	0.808	0.467
	420	24	3	0.698	0.046	0.027	20.133	2.150	1.241
	420	40	3	0.521	0.024	0.014	30.133	1.656	0.956
	420	60	3	0.582	0.032	0.019	26.267	1.877	1.084
	420	80	3	0.834	0.026	0.015	14.667	0.874	0.504
	425	24	3	0.690	0.045	0.026	20.500	2.100	1.212
	425	40	3	0.515	0.024	0.014	30.600	1.652	0.954
	425	60	3	0.578	0.033	0.019	26.467	1.966	1.135
	425	80	3	0.827	0.025	0.014	14.900	0.819	0.473
	430	24	3	0.682	0.044	0.025	20.867	2.101	1.213
	430	40	3	0.508	0.023	0.013	31.133	1.656	0.956
	430	60	3	0.575	0.031	0.018	26.667	1.815	1.048
	430	80	3	0.818	0.023	0.014	15.200	0.819	0.473
	435	24	3	0.674	0.043	0.025	21.233	2.050	1.184
	435	40	3	0.501	0.022	0.013	31.600	1.637	0.945
	435	60	3	0.570	0.028	0.016	26.933	1.701	0.982
	435	80	3	0.810	0.022	0.013	15.500	0.755	0.436
	440	24	3	0.666	0.041	0.024	21.600	2.000	1.155
	440	40	3	0.494	0.022	0.013	32.067	1.617	0.933
	440	60	3	0.565	0.027	0.015	27.267	1.665	0.961
	440	80	3	0.802	0.021	0.012	15.800	0.755	0.436
	445	24	3	0.660	0.039	0.023	21.933	2.001	1.155
	445	40	3	0.491	0.022	0.013	32.333	1.595	0.921
	445	60	3	0.564	0.025	0.015	27.367	1.595	0.921
	445	80	3	0.798	0.021	0.012	15.933	0.764	0.441
	450	24	3	0.659	0.039	0.022	21.967	1.904	1.099
	450	40	3	0.492	0.023	0.013	32.233	1.779	1.027
	450	60	3	0.566	0.026	0.015	27.200	1.609	0.929
	450	80	3	0.798	0.020	0.012	15.900	0.721	0.416
	455	24	3	0.656	0.038	0.022	22.167	1.904	1.099
	455	40	3	0.501	0.018	0.010	32.200	1.015	0.586
	455	60	3	0.565	0.025	0.014	27.233	1.553	0.897
	455	80	3	0.797	0.019	0.011	16.000	0.700	0.404
	460	24	3	0.651	0.037	0.021	22.400	1.852	1.069
	460	40	3	0.486	0.022	0.012	32.633	1.607	0.928
WL x TP	460	60	3	0.562	0.025	0.014	27.433	1.582	0.913
	460	80	3	0.791	0.018	0.010	16.233	0.643	0.371
	465	24	3	0.643	0.036	0.021	22.833	1.856	1.071
	465	40	3	0.480	0.020	0.012	33.167	1.550	0.895
	465	60	3	0.554	0.025	0.014	27.933	1.582	0.913
	465	80	3	0.779	0.018	0.010	16.633	0.635	0.367
	470	24	3	0.635	0.035	0.020	23.200	1.868	1.079
	470	40	3	0.473	0.019	0.011	33.700	1.493	0.862
	470	60	3	0.545	0.025	0.014	28.533	1.582	0.913
	470	80	3	0.767	0.019	0.011	17.133	0.723	0.418
	475	24	3	0.629	0.035	0.020	23.567	1.922	1.110

	475	40	3	0.468	0.019	0.011	34.033	1.436	0.829
	475	60	3	0.539	0.023	0.013	29.000	1.572	0.907
	475	80	3	0.759	0.018	0.010	17.433	0.737	0.426
	480	24	3	0.625	0.034	0.020	23.767	1.922	1.110
	480	40	3	0.466	0.018	0.010	34.267	1.380	0.797
	480	60	3	0.534	0.022	0.013	29.233	1.457	0.841
	480	80	3	0.753	0.019	0.011	17.633	0.737	0.426
	485	24	3	0.620	0.034	0.020	24.033	1.877	1.084
	485	40	3	0.463	0.018	0.010	34.467	1.380	0.797
	485	60	3	0.531	0.021	0.012	29.500	1.453	0.839
	485	80	3	0.748	0.019	0.011	17.867	0.777	0.448
	490	24	3	0.614	0.033	0.019	24.333	1.845	1.065
	490	40	3	0.457	0.018	0.010	35.000	1.418	0.819
	490	60	3	0.524	0.020	0.012	30.000	1.400	0.808
	490	80	3	0.739	0.019	0.011	18.233	0.833	0.481
	495	24	3	0.607	0.034	0.020	24.733	1.943	1.122
	495	40	3	0.450	0.017	0.010	35.500	1.308	0.755
	495	60	3	0.515	0.019	0.011	30.533	1.305	0.754
	495	80	3	0.727	0.021	0.012	18.767	0.874	0.504
	500	24	3	0.598	0.035	0.020	25.300	1.997	1.153
	500	40	3	0.441	0.016	0.010	36.233	1.332	0.769
	500	60	3	0.505	0.018	0.010	31.333	1.305	0.754
	500	80	3	0.716	0.017	0.010	19.233	0.808	0.467
	505	24	3	0.589	0.035	0.020	25.833	2.011	1.161
	505	40	3	0.431	0.017	0.010	37.100	1.389	0.802
	505	60	3	0.492	0.018	0.010	32.200	1.353	0.781
	505	80	3	0.698	0.024	0.014	20.100	1.114	0.643
	510	24	3	0.579	0.035	0.020	26.400	2.066	1.193
	510	40	3	0.423	0.016	0.009	37.733	1.328	0.767
	510	60	3	0.482	0.018	0.010	33.000	1.311	0.757
	510	80	3	0.684	0.024	0.014	20.700	1.114	0.643
	515	24	3	0.571	0.034	0.020	26.867	2.026	1.170
	515	40	3	0.413	0.018	0.010	38.600	1.587	0.917
	515	60	3	0.471	0.019	0.011	33.833	1.464	0.845
	515	80	3	0.674	0.023	0.013	21.200	1.114	0.643
WL x TP	520	24	3	0.566	0.032	0.019	27.267	2.026	1.170
	520	40	3	0.408	0.018	0.011	39.033	1.626	0.939
	520	60	3	0.466	0.019	0.011	34.233	1.504	0.869
	520	80	3	0.668	0.022	0.013	21.500	1.114	0.643
	525	24	3	0.561	0.033	0.019	27.600	2.042	1.179
	525	40	3	0.404	0.017	0.010	39.500	1.609	0.929
	525	60	3	0.461	0.019	0.011	34.667	1.557	0.899
	525	80	3	0.662	0.021	0.012	21.767	1.050	0.606
	530	24	3	0.555	0.033	0.019	27.900	2.042	1.179
	530	40	3	0.400	0.017	0.010	39.767	1.464	0.845
	530	60	3	0.456	0.019	0.011	35.067	1.557	0.899
	530	80	3	0.659	0.021	0.012	21.967	1.060	0.612

	535	24	3	0.551	0.032	0.019	28.100	2.042	1.179
	535	40	3	0.397	0.016	0.009	40.100	1.473	0.850
	535	60	3	0.453	0.019	0.011	35.300	1.552	0.896
	535	80	3	0.656	0.020	0.011	22.100	1.015	0.586
	540	24	3	0.549	0.031	0.018	28.267	2.026	1.170
	540	40	3	0.395	0.015	0.009	40.267	1.365	0.788
	540	60	3	0.447	0.019	0.011	35.467	1.557	0.899
	540	80	3	0.653	0.019	0.011	22.267	1.002	0.578
	545	24	3	0.551	0.031	0.018	28.233	1.986	1.146
	545	40	3	0.396	0.014	0.008	40.200	1.253	0.723
	545	60	3	0.452	0.020	0.012	35.367	1.656	0.956
	545	80	3	0.655	0.020	0.012	22.167	1.097	0.633
	550	24	3	0.548	0.030	0.018	28.400	1.947	1.124
	550	40	3	0.394	0.014	0.008	40.433	1.301	0.751
	550	60	3	0.449	0.022	0.013	35.567	1.801	1.040
	550	80	3	0.652	0.020	0.012	22.300	1.058	0.611
	555	24	3	0.543	0.029	0.017	28.700	1.873	1.082
	555	40	3	0.390	0.014	0.008	40.800	1.300	0.751
	555	60	3	0.445	0.024	0.014	35.900	1.900	1.097
	555	80	3	0.647	0.021	0.012	22.533	1.115	0.644
	560	24	3	0.540	0.028	0.016	28.933	1.795	1.037
	560	40	3	0.386	0.013	0.007	41.167	1.222	0.706
	560	60	3	0.442	0.024	0.014	36.167	1.950	1.126
	560	80	3	0.643	0.021	0.012	22.733	1.115	0.644
	565	24	3	0.535	0.028	0.016	29.233	1.818	1.049
	565	40	3	0.384	0.013	0.007	41.367	1.222	0.706
	565	60	3	0.440	0.025	0.014	36.333	2.101	1.213
	565	80	3	0.640	0.021	0.012	22.900	1.136	0.656
	570	24	3	0.532	0.027	0.016	29.400	1.778	1.026
	570	40	3	0.381	0.013	0.007	41.633	1.168	0.674
	570	60	3	0.437	0.025	0.014	36.567	2.103	1.214
	570	80	3	0.637	0.022	0.013	23.067	1.172	0.677
	575	24	3	0.529	0.026	0.015	29.600	1.778	1.026
	575	40	3	0.379	0.013	0.008	41.767	1.258	0.726
	575	60	3	0.435	0.025	0.014	36.733	2.101	1.213
WL x TP	575	80	3	0.634	0.023	0.013	23.267	1.250	0.722
	580	24	3	0.526	0.025	0.015	29.833	1.721	0.994
	580	40	3	0.377	0.013	0.008	42.033	1.266	0.731
	580	60	3	0.433	0.024	0.014	36.933	2.055	1.186
	580	80	3	0.631	0.022	0.013	23.400	1.217	0.702
	585	24	3	0.518	0.024	0.014	30.367	1.665	0.961
	585	40	3	0.372	0.014	0.008	42.533	1.266	0.731
	585	60	3	0.428	0.023	0.013	37.367	1.960	1.132
	585	80	3	0.622	0.022	0.013	23.867	1.250	0.722
	590	24	3	0.517	0.024	0.014	30.433	1.650	0.953
	590	40	3	0.372	0.013	0.007	42.533	1.168	0.674
	590	60	3	0.428	0.022	0.013	37.300	1.868	1.079

	590	80	3	0.622	0.023	0.013	23.900	1.217	0.702
	595	24	3	0.518	0.024	0.014	30.367	1.665	0.961
	595	40	3	0.373	0.012	0.007	42.367	1.159	0.669
	595	60	3	0.431	0.020	0.011	37.133	1.716	0.991
	595	80	3	0.624	0.023	0.013	23.767	1.242	0.717
	600	24	3	0.516	0.024	0.014	30.500	1.682	0.971
	600	40	3	0.371	0.012	0.007	42.567	1.102	0.636
	600	60	3	0.428	0.020	0.011	37.367	1.662	0.960
	600	80	3	0.621	0.022	0.013	23.967	1.242	0.717

WL: Wavelength (nm); TP: Temperature (°C); A: Absorbance (-); T: Transmittance (%); N: Number of replications; SD: Standard Deviation and SE: Standard Error.

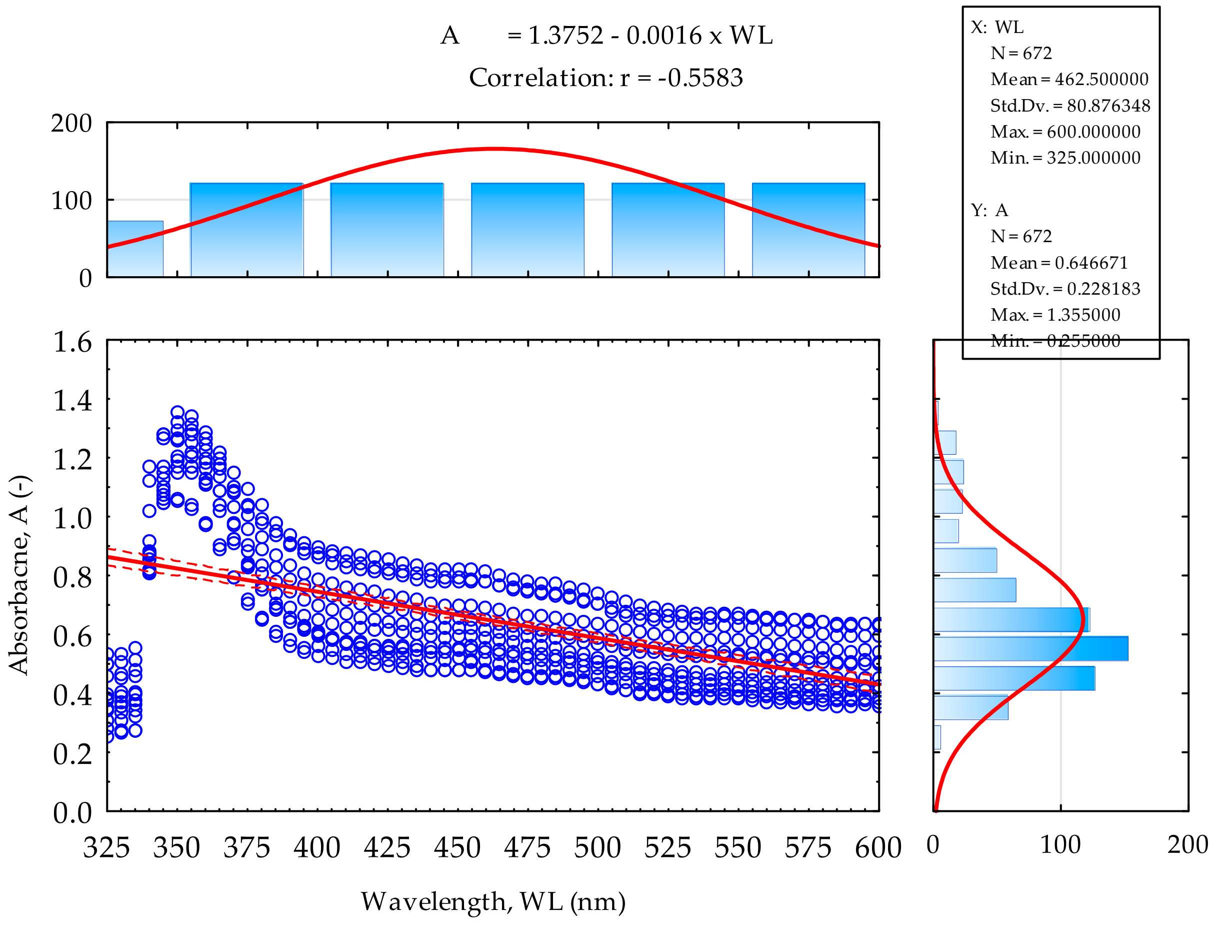

Figure S1. Scatterplot of absorbance versus wavelength at 95% confidence interval.

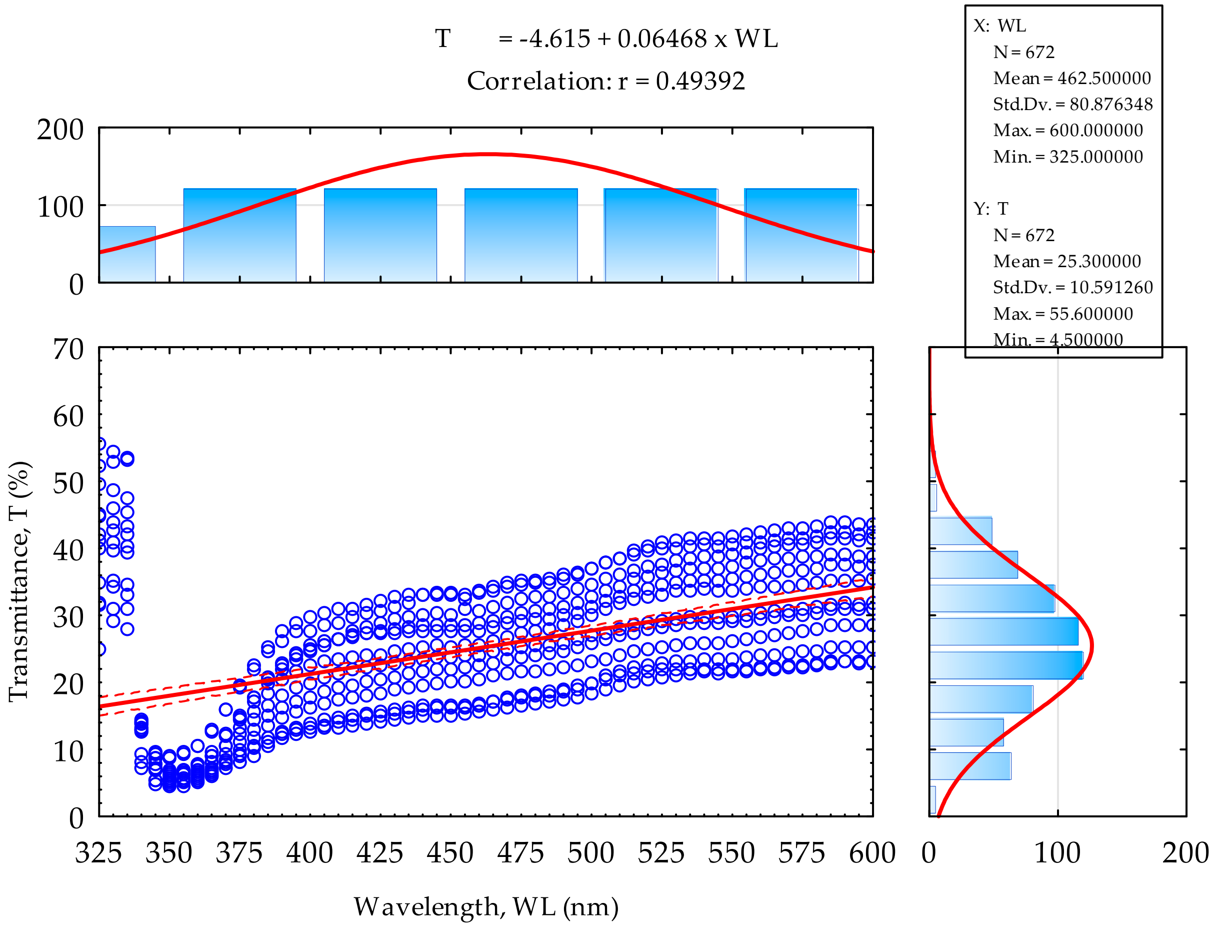

Figure S2. Scatterplot of transmittance versus wavelength at 95% confidence interval.

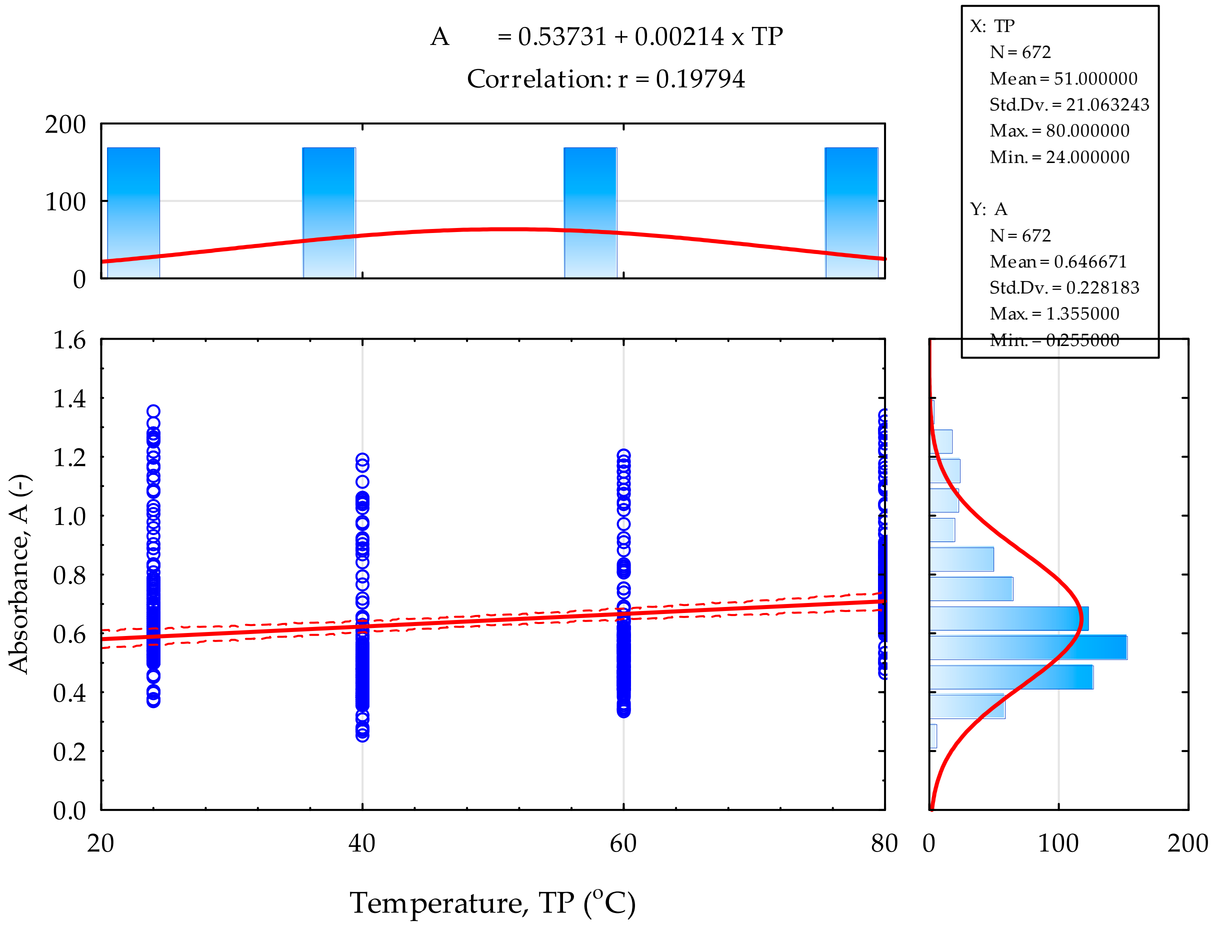

Figure S3. Scatterplot of absorbance versus temperature at 95% confidence interval.

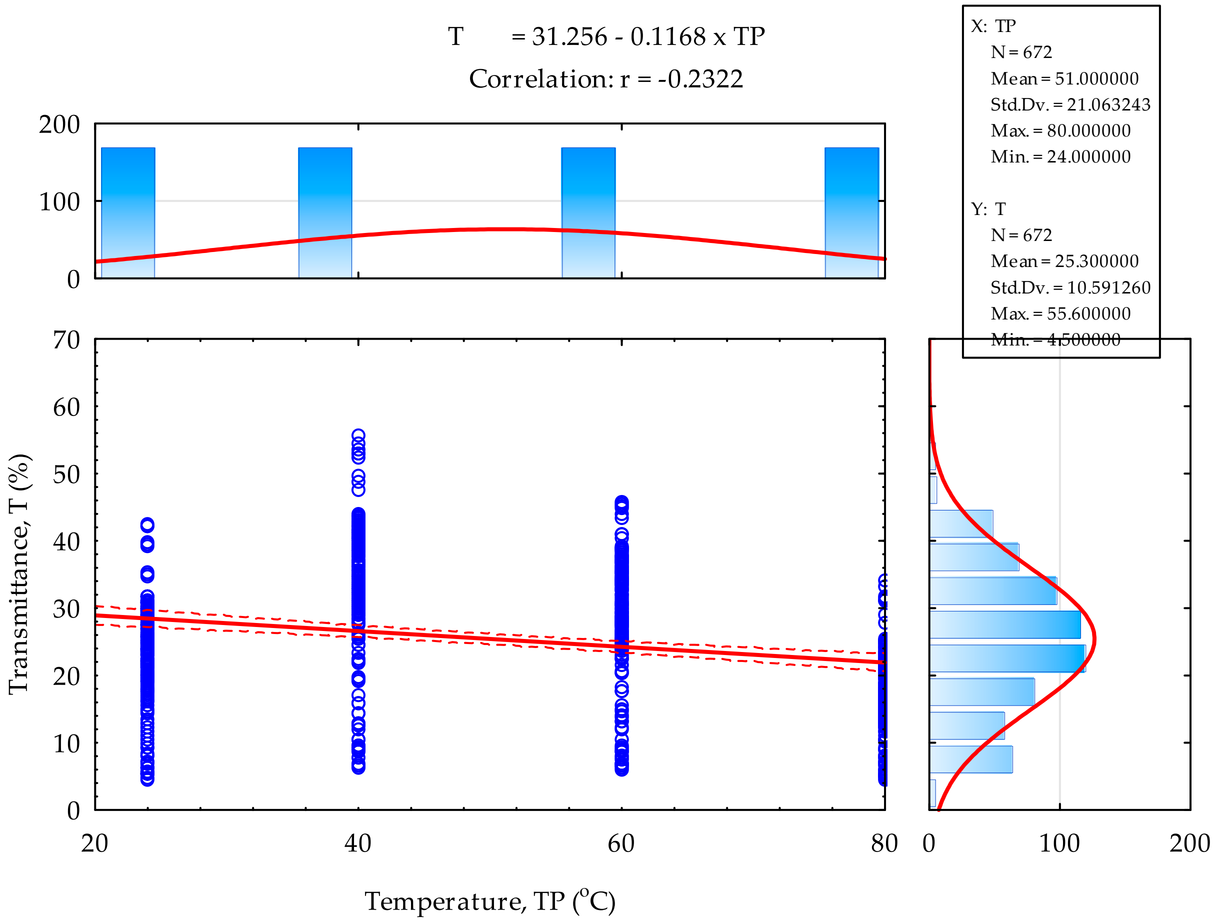

Figure S4. Scatterplot of transmittance versus temperature at 95% confidence interval.
